# Neutrophils undergo switch of apoptosis to NETosis during murine fatty liver injury via S1P receptor 2 signaling

**DOI:** 10.1038/s41419-020-2582-1

**Published:** 2020-05-18

**Authors:** Xinhao Zhao, Le Yang, Na Chang, Lei Hou, Xuan Zhou, Lin Yang, Liying Li

**Affiliations:** Department of Cell Biology, Municipal Laboratory for Liver Protection and Regulation of Regeneration, Capital Medical University, 100069 Beijing, China

**Keywords:** Innate immunity, Liver fibrosis, Experimental models of disease

## Abstract

Inappropriate neutrophil infiltration and subsequent neutrophil extracellular trap (NET) formation have been confirmed to be involved in chronic inflammatory conditions. Fatty liver disease is an increasingly severe health problem worldwide and currently considered the most common cause of chronic liver disease. Sphingosine 1-phosphate (S1P), a product of membrane sphingolipid metabolism, regulates vital physiological and pathological actions by inducing infiltration and activation of various cell types through S1P receptors (S1PRs). Here, we seek to determine the S1PR-mediated effects on neutrophil activation during chronic liver inflammation. In this study, NETs are detected in the early stage of methionine-choline-deficient and a high-fat (MCDHF) diet-induced liver injury. NET depletion by deoxyribonuclease I intraperitoneal injection significantly protects liver from MCDHF-induced liver injury in vivo. Meanwhile, we show that levels of myeloperoxidase-DNA complex (NET marker) in the serum present positive correlation with sphingosine kinase1 (S1P rate-limiting enzyme) messenger RNA expression or S1P levels in the injured liver of MCDHF-fed mice. In vitro, S1PR_2_ participates in the redirection of neutrophil apoptosis to NETosis via Gα_i/o_, extracellular signal-regulated kinase, p38 mitogen-activated protein kinase, and reactive oxygen species signaling pathways. Moreover, S1PR_2_ knockdown in MCDHF-fed mice by S1PR_2_-siRNA intravenous injection significantly inhibits NET formation in damaged liver tissue and then alleviates hepatic inflammation and fibrosis. Conclusion: In the early stage of fatty liver disease, S1PR_2_-mediated neutrophil activation plays an important role in the evolvement of liver injury.

## Introduction

Pathological sterile inflammation occurs during the development of many liver diseases, such as alcoholic steatohepatitis, nonalcoholic steatohepatitis (NASH), cholestatic hepatitis, drug-induced liver injury, and ischemia/reperfusion (I/R)^1–4^. The hepatocyte injury and Kupffer cell activation initiate a cytokine storm, which in turn propagates the recruitment of leukocytes, such as neutrophils^1,5,6^. Once liver injury happens, neutrophils infiltrate the site of injury within minutes in response to the release of damage-associated molecular patterns and their number peaks within hours and mediate the early responses to tissue damage^7^. However, most studies of neutrophils are performed using models of acute injury. Further research is needed before we can understand the role of inappropriate neutrophil activation in the development of chronic liver inflammatory diseases.

Traditionally, mature neutrophils are terminally differentiated with a brief circulating half-life of 6–8 h in humans and mice^8^. Constitutive neutrophil apoptosis is an essential mechanism for regulating neutrophil homeostasis. During inflammation, the longevity of neutrophil increases significantly by several folds as they become activated^9,10^. This issue is important, as activated neutrophils can perform complex duties and bring collateral damage to tissue. For instance, neutrophil extracellular trap (NET) formation, another form of cell death called NETosis, occurs in activated neutrophils^11–13^. Neutrophils stimulated by interleukin-8 (IL-8), lipopolysaccharide (LPS), phorbol myristate acetate (PMA), or bacteria undergo an oxidative burst and emit a network of DNA, histones, antibacterial, and potentially pro-inflammatory proteins^14^, while peptidylarginine deiminase 4-mediated histones’ citrullination and chromatin decondensation are essential steps in this progress^15,16^. Initially, NETs have been described as a beneficial mechanism of host defense against pathogens. However, it has been recently regarded as a harmful contributor in various sterile inflammatory conditions, including atherosclerosis, venous thrombosis, lung injury, tumor metastasis, and liver inflammation^17,18^. Therefore, it is necessary to clarify the mediator and underlying molecular mechanism of regulating neutrophil function and develop therapeutic strategies for the prevention and treatment of liver inflammatory diseases.

Sphingosine 1-phosphate (S1P), one of the crucial regulators in physiological and pathophysiological processes, is formed through phosphorylation of sphingosine in a reaction catalyzed by two isoforms of sphingosine kinase (SphK), SphK1 and SphK2^19^. S1P is an agonist of five specific G protein-coupled S1P receptors (S1PR)_1–5_ that activate diverse downstream signaling pathways. Additionally, S1P has been confirmed to regulate diverse cellular processes, which are important for inflammation responses^20^. Our previous studies have shown that hepatic S1P levels are dramatically increased and play an important role in the exacerbation of liver injury caused by multiple etiologies^21–23^. However, the role of S1P in neutrophil function during chronic liver inflammation remains incompletely defined.

Our findings suggest that neutrophils play an important role in the initial period of chronic liver inflammation through NET formation. S1PR_2_ mediates the switch of apoptosis to NETosis in vitro, which is dependent on Gα_i/o_, extracellular signal-regulated kinase (ERK), p38, and reactive oxygen species (ROS) signaling pathways. Furthermore, S1PR_2_ knockdown ameliorates liver inflammation and fibrosis by inhibiting NET formation in vivo, which may represent an effective therapeutic strategy for fatty liver diseases.

## Materials and methods

### Mouse models

ICR male mice aged 6 weeks were fed methionine-choline-deficient and a high-fat (MCDHF) diet (Research Diet, New Brunswick, NJ, USA) as described previously^23^. Mice were sacrificed at day 1, 3, 7, 14, 28, or 56 after MCDHF treatment. To eliminate NETs in vivo, we treated mice with deoxyribonuclease I (DNase I). The intraperitoneal injection of DNase I (50 μg/mouse, Roche) or vehicle was performed 24 h before feeding an MCDHF diet, and then three times per week. S1PR_2_ knockdown mouse model was constructed by intravenous injection of chemically modified small interfering RNA (siRNA) of S1PR_2_ (UAA CUC CCG UGC AGU GGU UUU) purchased from Thermo Scientific (Lafayette, CO, USA). Control mice were injected with an equal volume of scramble (SCR) siRNA dissolved in phosphate-buffered saline (PBS). S1PR_2_-siRNAs were injected one day before MCDHF-induced liver injury, and then twice every week. Based on our published studies, six mice per group were used for the animal studies^23–25^. The animal studies were randomized and blinded according to the protocol approved by the Ethics Committee of Capital Medical University and in accordance with the approved guidelines (approval number: AEEI-2017-090).

### BM transplantation

ICR male mice aged 6 weeks received lethal irradiation (8 Gy) and immediately received transplantation by a tail vein injection of 1.5 × 10^7^ whole bone marrow (BM) cells obtained from 3-week-old enhanced green fluorescence protein (EGFP) transgenic mice. Four weeks later, mice whose BM was rebuilt were subjected to MCDHF-induced liver injury.

### Fluorescence-activated cell sorting

Nonparenchymal cells of mouse liver were isolated as described previously^23^. APC-Ly6G (BD Bioscience, Franklin Lakes, NJ, USA) and its isotype-matched negative control were added to the nonparenchymal cell suspension, respectively. After 15 min incubation in the dark, the cells were washed with PBS and subjected to fluorescence-activated cell sorting (FACS), which was performed on a FACSAria and analyzed with FACS Diva 4.1 (BD Biosciences).

### Isolation of mouse BM neutrophils

BM cells, acquired from ICR mice aged 6 weeks as described^25^, were layered in a ratio of 1:3 on top of Histopaque 1077 (Sigma-Aldrich, St. Louis, MO, USA), after centrifugation, and the precipitate was resuspended with PBS. The cell suspension was layered in a ratio of 1:2 on top of Histopaque 1119 (Sigma-Aldrich), after centrifugation, and neutrophils were recovered on the top of Histopaque 1119^26^. Neutrophils were washed with PBS and then resuspended in RPMI medium 1640.

### Detection of apoptosis

The Annexin V/PI apoptosis detection kit (BD Biosciences) was used to detect apoptosis according to the manufacturer’s instructions. Caspase-3 cleavage detected by western blot analysis was also used as additional indicators of apoptosis.

### MPO-DNA complex detection

A capture enzyme-linked immunosorbent assay (ELISA) detecting MPO associated with DNA (MPO-DNA) was performed as described^27^. For the capture antibody, an MPO ELISA kit (Hycult; HK210-01) and a peroxidase-labeled anti-DNA monoclonal antibody (component 2, Cell Death ELISAPLUS; Roche) were used according to the manufacturer’s directions.

### Western blot analysis

Western blot analysis was performed with 50 μg of protein extract from cells or 100 μg from liver tissue, using monoclonal antibodies: citrullinated-histone H3 (1:1000, Abcam, ab5103); ERK1/2(1:1000, Cell Signaling, 4695S) and phosphor-ERK1/2 (1:1000, Cell Signaling, 4376S), p38 (1:1000, Cell Signaling, 9212S), and phosphor-p38 (1:1000, Cell Signaling, 9211S); caspase-3 (1:1000, Cell Signaling, 9662S) and cleaved caspase-3 (1:1000, Cell Signaling, 9661S); anti-β-tubulin (1:5000, Cell Signaling, 2146S) and anti-β-actin monoclonal antibodies (1:5000, Cell Signaling, 3700S). ODYSSEY goat anti-rabbit IRDye® 800 CW antibody and goat anti-mouse IRDye® 800 CW antibody (1:10,000, LI-COR) were used as secondary antibodies. The bands were displayed using ODYSSEY and quantified by Odyssey v3.0 software. β-Tubulin or β-actin was used as reference.

### Immunofluorescence staining

Cells were fixed in 4% paraformaldehyde in PBS for 30 min and permeabilized in 0.5% Triton X-100 in PBS for 3 min; after blocking with 2% bovine serum albumin (Roche), they were incubated with the specific primary antibodies for S1PR_1_ (1:50, Santa Cruz, sc-25489); S1PR_2_ (1:50, Santa Cruz, sc-25491); S1PR_3_ (1:50, Santa Cruz, sc-30024); citrullinated-histone H3 (Cit-H3) (1:200, Abcam), myeloperoxidase (MPO) (1:200; Abcam, 14569), or neutrophil elastase (NE) (1:100, Abcam, ab21595), and secondary antibody conjugated with Cy3 (1:100, Jackson Immunoresearch) was a secondary antibody. The samples were observed under a confocal microscope (LSM510, Carl Zeiss MicroImaging). Fluorescence intensity and NET formation were analyzed by ImageJ 1.4 (NIH).

### RT-qPCR

Total RNA was extracted from cells or liver frozen specimen using RNeasy mini kit (Qiagen, Germany) and the quantity and purity of RNA was determined by NanoDrop 2000 spectrophotometer (Thermo Fisher Scientific). Complementary DNA was synthesized using oligo (dT) and M-MLV reverse transcriptase (Invitrogen). Quantitative reverse transcription-PCR (RT-qPCR) was performed using SYBR Green qPCR Master Mix on the ABI 7300 TH Real-Time PCR System (Applied Biosystems). All primers were synthesized by Biotech (Beijing, China). Primers used for RT-qPCR were as follows: *Rn18s*: sense, 5′-GTA ACC CGT TGA ACC CCA TT-3′; antisense, 5′-CCA TCC AAT CGG TAG TAG CG-3′. *Ly6g*: sense, 5′-AGA AGCA AAG TCA AGA GCA ATC TCT-3′; antisense, 5′-TGA CAG CAT TAC CAG TGA TCT CAG T-3′; *Sphk1*: sense: 5′- TGT CAC CCA TGA ACC TGC TGT CCC TGC ACA-3′; antisense: 5′-AGA AGG CAC TGG CTC CAG AGG-3′. *S1pr1*: sense: 5′-ACT TTG CGA GTG AGC TG-3′; antisense: 5′-AGT GAG CCT TCA GTT ACA GC-3′. *S1pr2*: sense: 5′-TTC TGG AGG GTA ACA CAG TGG T-3′; antisense, 5′-ACA CCC TTT GTA TCA AGT GGC A-3′. *S1pr3*: sense: 5′-TGG TGT GCG GCT GTC TAG TCA A-3′; antisense, 5′-CAC AGC AAG CAG ACC TCC AGA-3′. *Tnf*: sense, 5′-GGC AGG TTC TGT CCC TTT CA-3′; antisense, 5′-CTG TGC TCA TGG TGT CTT TTC TG-3′. *Acta2*: sense: 5′-ATG CTC CCA GGG CTG TTT T-3′; antisense: 5′-TTC CAA CCA TTA CTC CCT GAT GT-3′. *Col1a1*: sense: 5′-AGG GCG AGT GCT GTG CTT T-3′; antisense: 5′-CCC TCG ACT CCT ACA TCT TCT GA-3′. *Col3a1*: sense: 5′-TGA AAC CCC AGC AAA ACA AAA-3′; antisense, 5′-TCA CTT GCA CTG GTT GAT AAG ATT AA-3′. *Il6*: sense, 5′-CTC TGG GAA ATC GTG GAA ATG-3′; antisense, 5′-AAG TGC ATC ATC GTT GTT CAT ACA-3′. *Ilb*: sense, 5′-GCA ACT GTT CCT GAA CTC AAC T-3′; antisense, 5′-ATC TTT TGG GGT CCG TCA ACT-3′.

### ROS production

2′,7′-Dichlorofluorescein diacetate (DCFDA) (Sigma-Aldrich) is a cell-permeable, non-fluorescent probe; it is de-esterified intracellularly and turns to highly fluorescent 2′,7′-dichlorofluorescein upon oxidation. BM neutrophils were incubated with DCFDA for 20 min, and after seeding in 96-well plates, they were treated with S1P. The plate was then transferred onto a fluorescent plate reader, EnVision 2104-0010 (PerkinElmer, MA, USA), and detected the fluorescent value.

### S1P quantitation

The concentration of hepatic S1P was measured by the S1P ELISA kit (Echelon, USA) according to the manufacturer’s instructions. A standard curve was created, and the results were normalized to the protein content of the sample (pmol S1P/mg protein).

### Liver damage assessment

Serum alanine aminotransferase (ALT) levels were detected by BS-200 Chemistry Analyzer (Mindray, China). Serum γ-glutamyl transpeptidase (GGT) levels were determined by a GGT ELISA kit (Cusabio, China) according to the manufacturer’s directions.

### Histology analysis

Liver tissues were fixed in 4% buffered formaldehyde. Liver tissue sections (5 μm) were stained with hematoxylin and eosin (H&E) staining for assessment of inflammation and injury and Sirius Red staining for the extent of collagen deposition. Morphometric analysis was conducted with researcher blind to the treatment. We measured 15 randomly selected areas per sample and calculated the mean value of the percentage of inflammatory or fibrosis area accounting for total area. Morphometric analysis of H&E and Sirius Red staining was done using ImageJ 1.4 (NIH).

### Statistical analysis

The results were expressed as mean ± standard error of the mean (SEM). Comparisons between two independent groups were performed using a two-sample *t* test. Comparisons between multiple groups were performed by one- or two-way ANOVA (analysis of variance) with post hoc Tukey’s multiple comparison tests when appropriate. Correlation coefficients were calculated by Pearson’s test. *P* < 0.05 was considered to be significant. All in vitro experiments were executed duplicable and repeated three times. The animal experiments were conducted with six mice in each group.

## Results

### Significant neutrophil infiltration and NET formation occur in the liver of MCDHF-fed mice

**Fig. 1:**
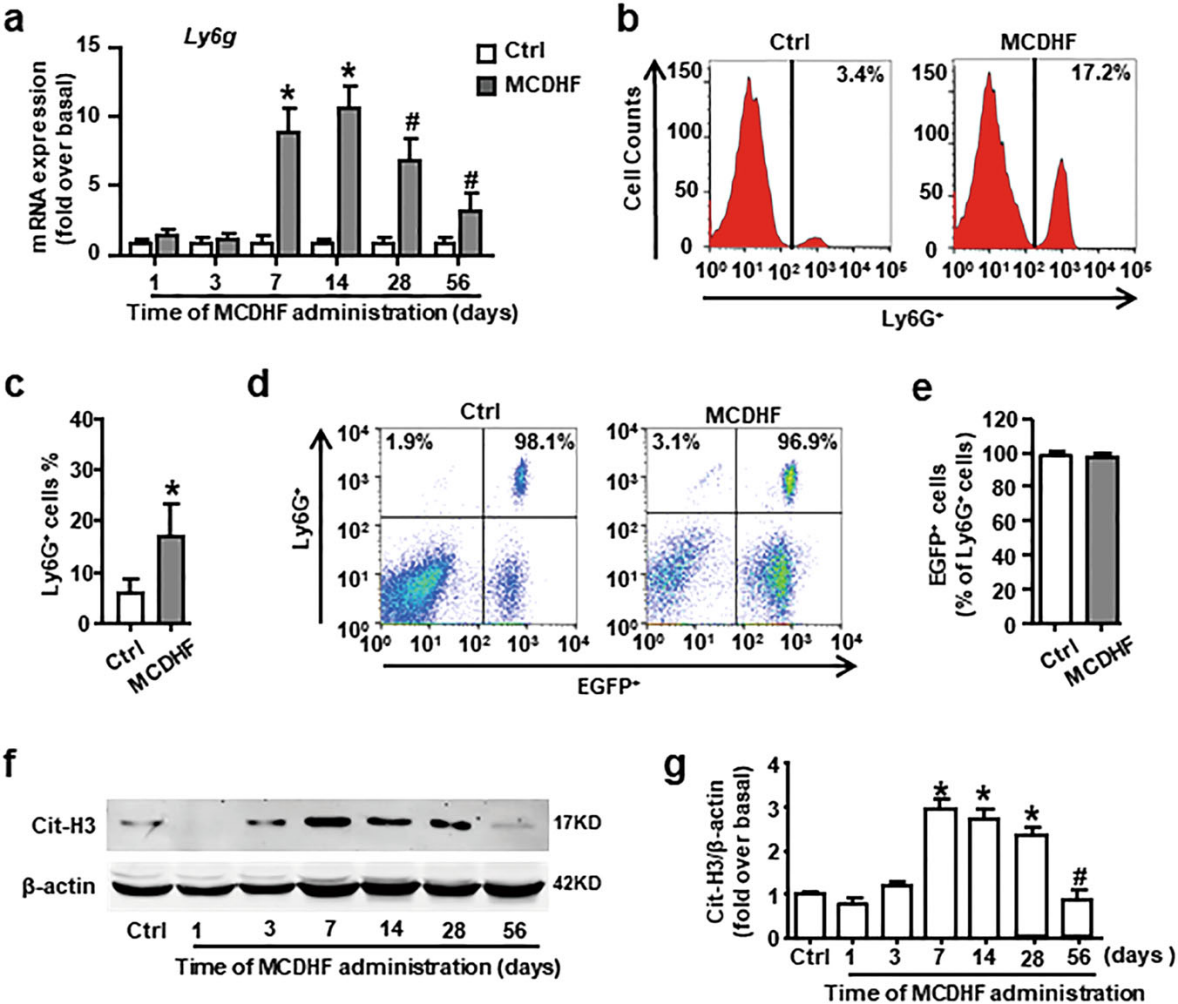
Excessive BM-derived neutrophils accumulate and undergo NETosis in the liver of MCDHF-fed mice. **a** The mRNA expression of neutrophil marker *Ly6g* was examined by RT-qPCR in the injured liver of MCDHF-treated mice (*n* = 6 per group). **b**, **c** Representative FACS plots and quantification for total neutrophils (Ly6G^+^). **d**, **e** Representative FACS plots and quantification for neutrophils of BM origin (Ly6G^+^ EGFP^+^). **f**, **g** Cit-H3 protein levels in the injured liver were examined by western blot. Data are presented as the mean ± SEM. Comparisons between two independent groups were performed using a Student’s *t* test. One-way ANOVA was used in **a**, **g**. *P < 0.05 vs. control. ^*#*^*P* < 0.05 vs. MCDHF-treated group for 14 days.

At first, we investigated the dynamic change of neutrophil marker *Ly6g* messenger RNA (mRNA) expression in the liver of mice treated with MCDHF diet at different time points. The results showed that *Ly6g* mRNA expression was upregulated from 7 days after treated with MCDHF diet (Fig. 1a), and then dramatically decreased after 14 days. Further, FACS analysis revealed that the percentage of Ly6G^+^ neutrophils (control group: 6.40%; MCDHF group: 17.22%) in nonparenchymal cells was elevated obviously at 14 days in the liver of MCDHF-treated mice (Fig. 1b, c). These data indicate that numerous neutrophils are recruited to injured liver and participate in the early stage of inflammatory response during chronic liver injury.

To clarify the origin of neutrophils recruited to the injured liver, we performed a genetic EGFP-labeled BM cell transplantation to the mice that had been lethally irradiated. Then, the chimeric mice received MCDHF diet for 14 days. We isolated hepatic nonparenchymal cells from the liver tissue and detected Ly6G^+^ cells by FACS. Among Ly6G^+^ cells, the percentage of EGFP^+^ cells nearly reached 100% in each group (Fig. 1d, e), indicating that the recruited neutrophils were mostly derived from the BM in the damaged liver.

We then examined the activities of the infiltrated neutrophils. An increased protein expression of Cit-H3, a specific marker of NETs, was detected in the liver of mice treated with MCDHF diet for 7 days. Similar to *Ly6g* mRNA expression in the liver, Cit-H3 expression gradually decreased after 28 days and returned to baseline at 56 days (Fig. 1f, g), implying that neutrophils were activated to undergo NETosis during the initial period. Altogether, these results demonstrate that BM neutrophil recruitment and NET formation occur in the early stage of fatty liver injury.

### Depletion of NETs by DNase I protects liver from MCDHF-induced injury

**Fig. 2:**
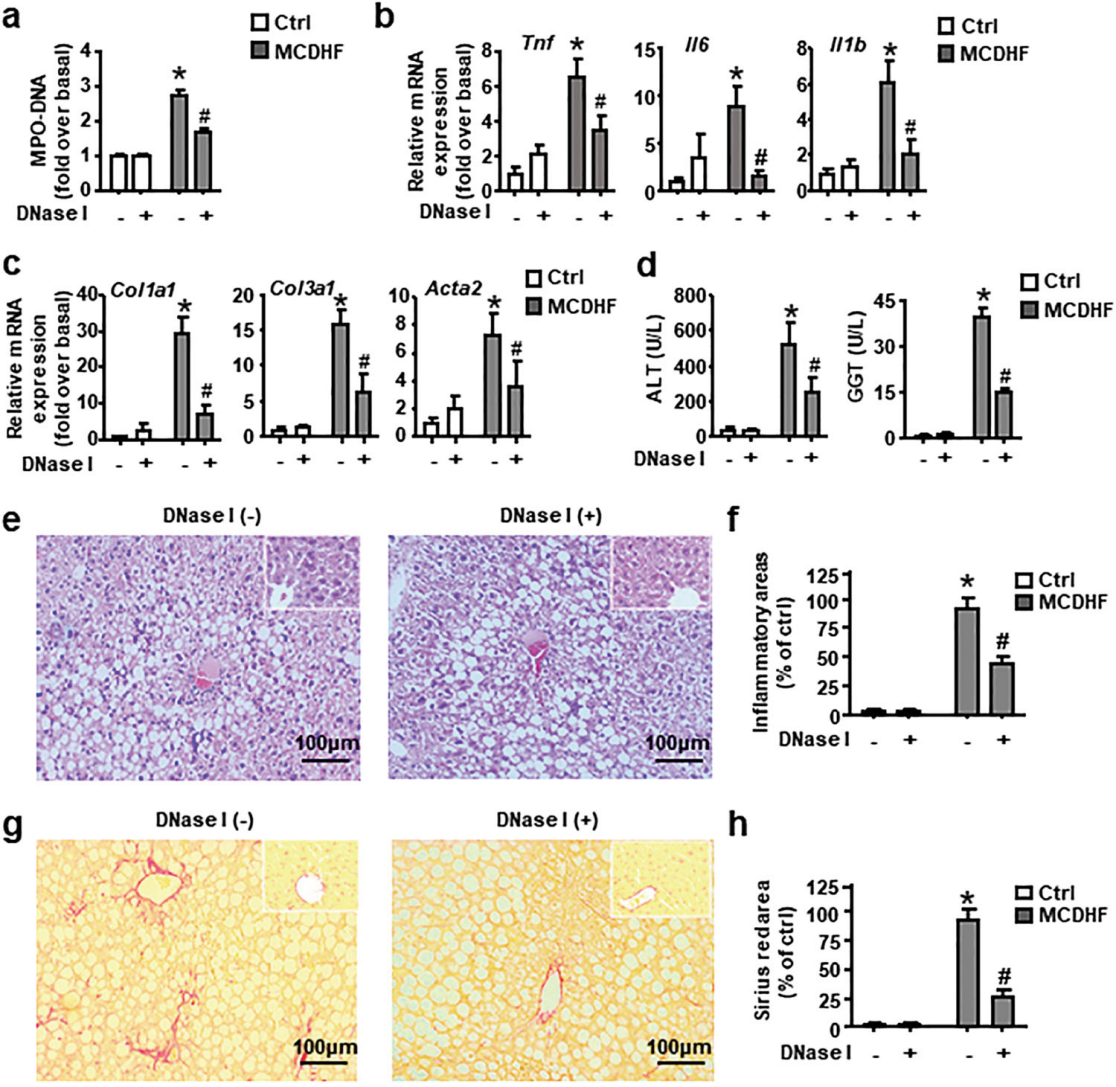
Depletion of NETs by DNase I mitigate liver injury in MCDHF-fed mice. **a** Serum MPO-DNA levels were assessed in mice treated with PBS or DNase I with or without MCDHF diet (*n* = 6 per group). **b** Hepatic mRNA expression of inflammatory cytokines: *Tnf*, *Il6*, and *Il1b*, **c** mRNA expression of liver fibrosis hallmarks: *Col1a1*, *Col3a1*, and *Acta2* and **d** ALT and GGT levels in the serum were determined in mice treated with MCDHF diet or control diet in the presence or absence of DNase I administration. **e**, **f** Representative images of H&E-stained or **g**, **h** Sirius Red-stained liver sections from mice treated with MCDHF diet for 2 weeks with or without DNase I administration. Inset: H&E or Sirius Red staining for control-diet group in the presence or absence of DNase I administration. Scale bars, 100 µm. Inflammatory and fibrosis areas were quantified. Data are presented as the mean ± SEM. Two-way ANOVA was used. *P < 0.05 vs. Ctrl. ^#^*P* < 0.05 vs. MCDHF-treated alone.

Next, we assessed the key role of neutrophil activation in chronic liver inflammation. We manipulated an intraperitoneal injection of DNase I to disrupt the DNA strands comprising the structure of NETs^28^. Circulating MPO-DNA complexes (NETs marker) were significantly reduced in the serum (Fig. 2a), demonstrating the efficacy of DNase I at depletion of NETs. Simultaneously, the depletion of NETs resulted in a significant reduction in hepatic mRNA expression of pro-inflammatory mediators, ALT and GGT levels in the serum, and mRNA expression of fibrosis hallmarks (Fig. 2b–d). In line with RT-qPCR results, H&E and Sirius Red staining showed that inflammation and collagen deposition were markedly attenuated (Fig. 2e–h). In sum, these data provide evidence that elimination of NET formation protects the liver from MCDHF-induced liver damage.

### Hallmarks of neutrophil activation correlate positively with SphK1/S1P in the MCDHF-fed mice

**Fig. 3:**
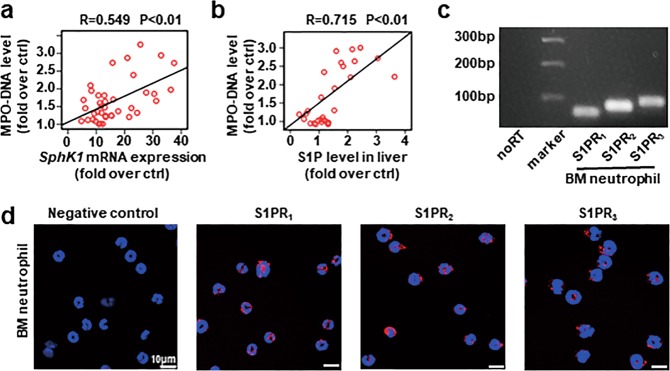
S1P plays a positive role in the activation of neutrophils. **a** The correlation between MPO-DNA complex level in the serum and hepatic *SphK1* mRNA expression or **b** S1P level in the liver of MCDHF-fed mice was analyzed by regression analysis. c The RT-PCR production of S1PR_1/2/3_ was size-fractionated in a 2% agarose gel. **d** Representative images of immunofluorescent staining for S1PR_1/2/3_ (red) in mouse BM neutrophil. The nuclei were stained with DAPI (blue). Scale bars, 10 µm. Pearson’s test was used in **a**, **b**.

Elevated *Sphk1* expression and S1P concentration are detected in the liver of mouse models and patients in our previous reports^22,23,29,30^. Here we undertook correlation analysis between *Sphk1* mRNA expression or S1P levels and MPO-DNA levels in MCDHF-fed mice. We discovered that hepatic *Sphk1* expression positively correlated with MPO-DNA levels (Fig. 3a). Meanwhile, there existed a positive correlation between hepatic S1P concentration and amount of MPO-DNA complex in the serum (Fig. 3b). These data suggest that S1P may play an important role in the activation of neutrophils during MCDHF-induced liver injury.

### S1PR_2_ mediates NETosis induced by S1P in vitro

First of all, S1PR_1–3_ expression in BM neutrophil was detected at the mRNA level by agarose gel electrophoresis of reverse transcription-PCR products (Fig. 3c), and the protein level by immunofluorescence (Fig. 3d) in vitro.

**Fig. 4:**
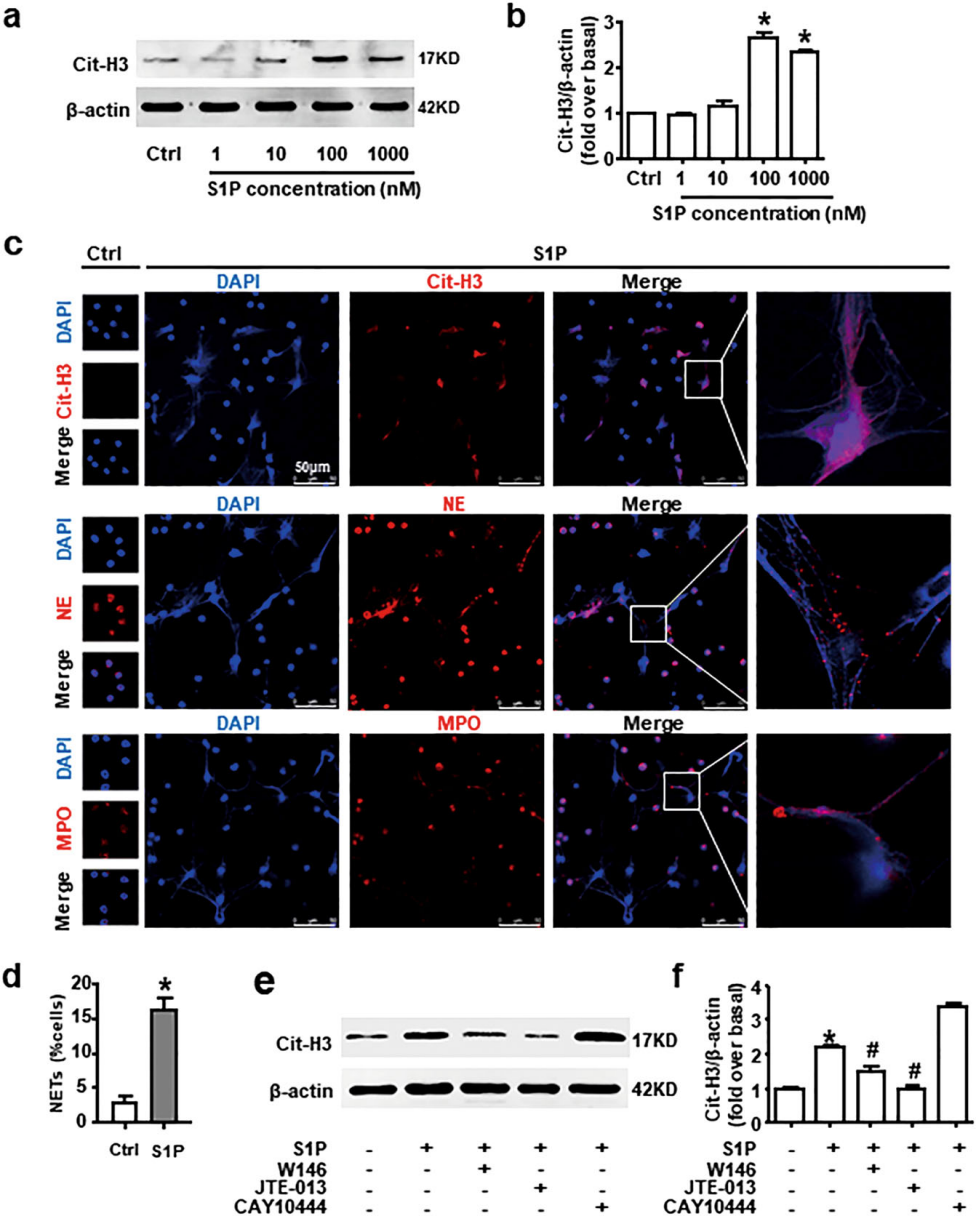
S1P-induced NETosis is mediated by S1PR_2_ in vitro. **a**, **b** Protein expression of Cit-H3 treated with S1P gradients of 1, 10, 100, and 1000 nM for 4 h was measured by western blot. **c** Representative images of Cit-H3, NE, or MPO (red) immunofluorescent staining in S1P-stimulated neutrophils. The nuclei were stained with DAPI (blue). Scale bars, 50 µm. **d** Quantification of neutrophils undergo NETosis with or without S1P treatment. **e**, **f** S1P-induced Cit-H3 protein level with or without inhibitors of S1PR_1/2/3_ pretreatment was examined by western blot. Data are presented as the mean ± SEM (*n* = 6 per condition in three experiments). One-way ANOVA was used in **b**. A Student’s *t* test was used in **d**. Two-way ANOVA was used in **f**. *P < 0.05 vs. control. ^#^*P* < 0.05 vs. S1P-treated alone.

Considering the positive correlation between SphK1/S1P and NET biomarker, MPO-DNA, we tested whether S1P contributed to NETosis in vitro. The results of western blot analysis revealed that S1P facilitated Cit-H3 expression in neutrophil in a dose-dependent manner (Fig. 4a, b). In addition, NETosis induced by S1P was confirmed by immunofluorescence confocal microscopy and quantitative analyses (Fig. 4c, d). We observed the presence of manifest web-like chromatin release, in which chromatin [DAPI (4′,6-diamidino-2-phenylindole), blue] had good co-location with NE (red), MPO (red), and Cit-H3 (red), respectively. Cit-H3 was specially expressed in neutrophils underwent NETosis. To explore which S1PR subtypes were involved in this progress, the specific S1PR antagonists were employed. Instead of CAY10444, W146, or JTE-013 inhibited S1P-induced Cit-H3 expression, while the latter one could totally block this process (Fig. 4e, f). These data implicate that S1PR_2_ is necessary for the regulation of NETosis induced by S1P.

### S1PR_2_ knockdown obviously attenuates NET formation in MCDHF-induced liver injury

**Fig. 5:**
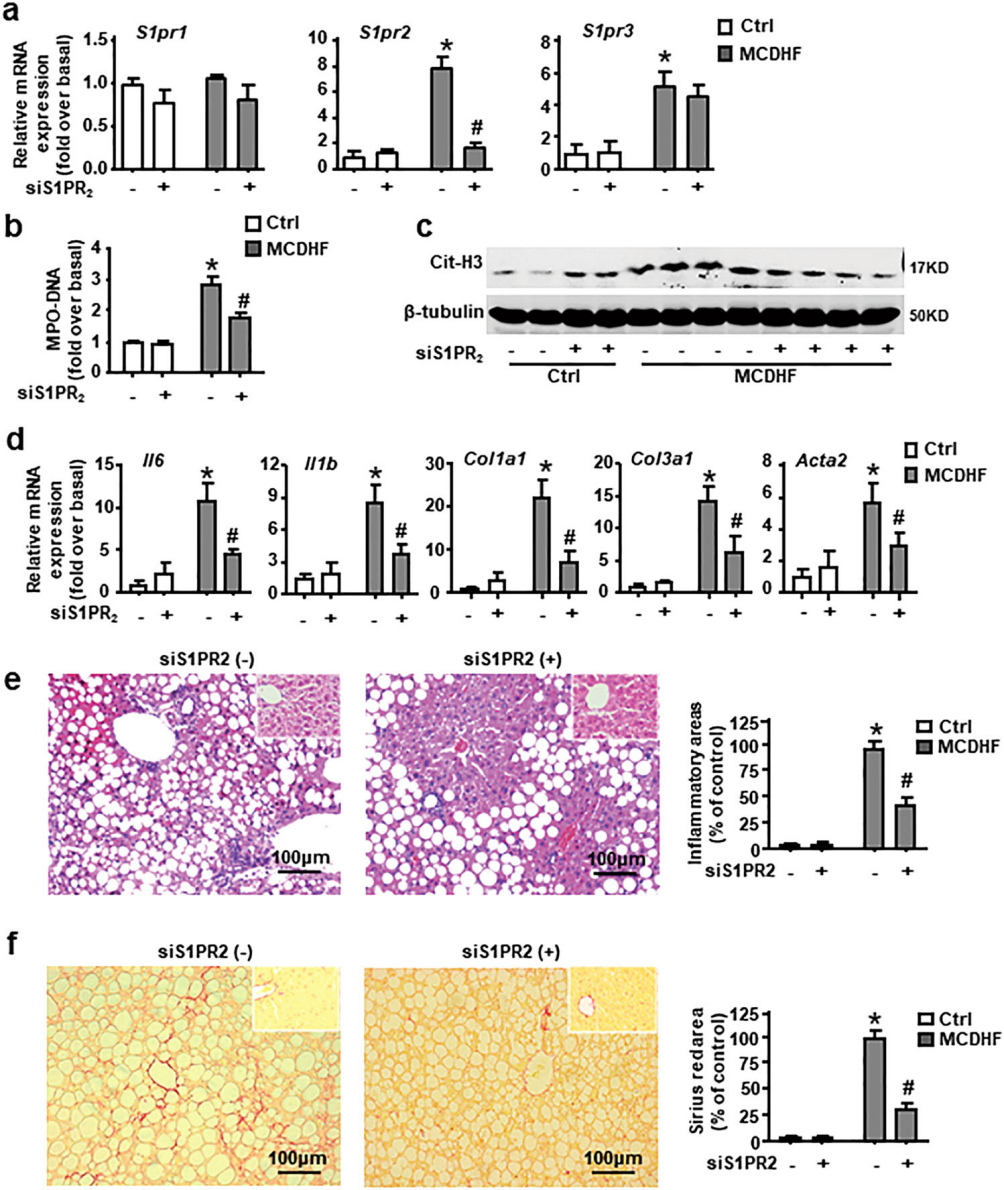
S1PR_2_-siRNA reduced NET formation in liver of MCDHF-treated mice. **a** Effectiveness of S1PR_2_-siRNA in vivo was measured by RT-qPCR. **b** MPO-DNA levels in the serum, **c** hepatic Cit-H3 protein level, and **d** mRNA expression of inflammatory cytokines: *Il6*, *Il1b* and fibrosis hallmarks: *Col1a1*, *Col3a1*, and *Acta2* were determined by RT-qPCR in the liver of mice treated with control diet or MCDHF diet in the presence or absence of S1PR_2_-siRNA administration. **e**, **f** Representative images of H&E- or Sirius Red-stained liver sections after 2 weeks MCDHF feeding with or without S1PR_2_-siRNA administration. Inset: H&E or Sirius Red staining for control-diet group in the presence or absence of S1PR_2_-siRNA administration. Inflammatory and fibrosis areas were quantified. Scale bars, 100 µm. Data are presented as the mean ± SEM (*n* = 6 per group). Two-way ANOVA was used. *P < 0.05 vs. Ctrl. ^*#*^*P* < 0.05 vs. MCDHF-treated alone.

In vitro, blockade of S1PR_2_ inhibits NETosis induced by S1P. To determine whether S1PR_2_ mediated neutrophil activation in vivo, we subjected mice to intravenous injection of S1PR_2_-siRNA. S1PR_2_-siRNA markedly and specially downregulated *S1pr2* mRNA expression in the liver of MCDHF-fed mice (Fig. 5a). S1PR_2_ blockage significantly decreased the levels of MPO-DNA complex in the serum and protein levels of Cit-H3 in the injured liver (Fig. 5b, c). Moreover, mRNA expression of inflammatory cytokines (*Tnf*, *Il6*, and *Il1b*) and fibrosis hallmarks (*Col1a1*, *Col3a1*, *and Acta2*) were obviously reduced in the injured liver (Fig. 5d). Simultaneously, in accordance with RT-qPCR results, histologic analysis exhibited that liver inflammation and fibrosis were predominantly attenuated under S1PR_2_-siRNA administration (Fig. 5e, f). Taken together, these results prove that S1PR_2_ knockdown notably alleviates MCDHF-induced liver injury via inhibition of NET formation.

### S1PR_2_ mediates inhibition of neutrophil spontaneous apoptosis induced by S1P

**Fig. 6:**
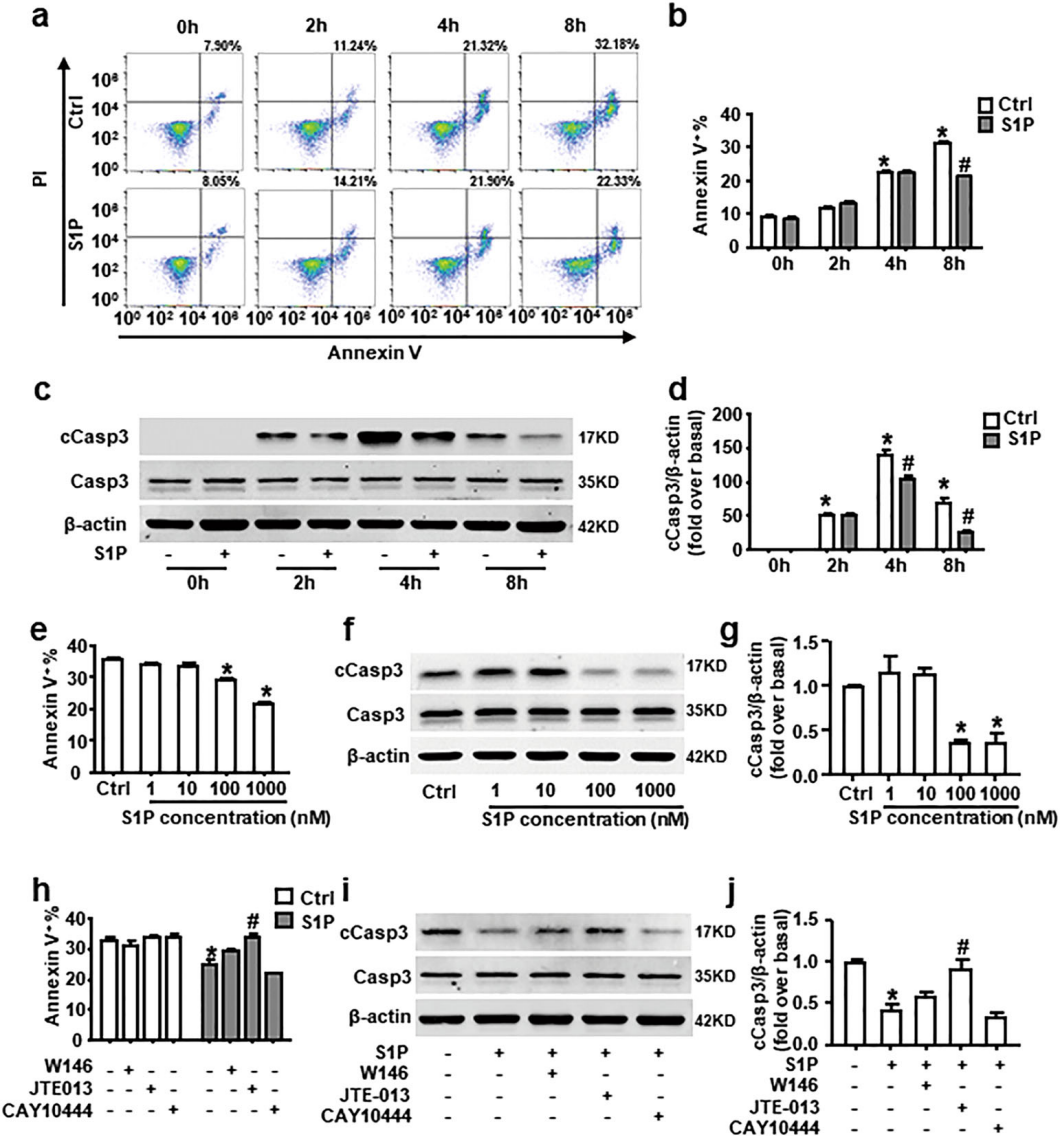
S1P delays neutrophil spontaneous apoptosis dependent on S1PR_2_ in vitro. The apoptotic rate of isolated neutrophils was measured by Annexin V and PI staining. The percentage of apoptotic neutrophils (Annexin V^+^) is shown. Protein expression of cCasp3 and Casp3 was measured by western blot. **a**–**d** BM neutrophils were cultured with or without S1P for 0, 2, 4, or 8 h. A representative flow cytometric plot of Annexin V and PI staining of a single sample from each group was provided. **e**–**g** Neutrophils were treated with S1P gradients of 1, 10, 100, and 1000 nM for 8 h. **h**–**j** Cells were pre-incubated with W146, JTE-013, or CAY10444 for 30 min, and then stimulated with S1P (1 µM) for 8 h. Data are presented as the mean ± SEM (*n* = 6 per condition in three experiments). One-way ANOVA was used in **e**, **g**, and two-way ANOVA was used in **b**, **d**, **h**, **j**. *P < 0.05 vs. control. ^#^*P* < 0.05 vs. S1P-treated alone.

Under inflammatory conditions, diverse stimuli such as pro-inflammatory cytokines or bacterial components can prolong neutrophil lifespan and exaggerate neutrophil-induced inflammation^11,31–33^. Over the years, studies in many different cell types have outlined that S1P is implicated in suppression of apoptosis^19,20^. We speculated that S1P could switch neutrophil spontaneous apoptosis to NETosis. First, we evaluated apoptosis in freshly prepared neutrophils by measuring the percentage of Annexin V^+^ cells and protein expression of cleaved caspase-3 (cCasp3). Similar to the previously published paper^9,10,34^, we observed that ~24% of neutrophils underwent apoptosis after 4 h and the percentage of Annexin V^+^ cells were increased with time (Fig. 6a, b). Meanwhile, the elevated expression of cCasp3 was detected (Fig. 6c, d). Whereas the percentage of apoptotic cell and cCasp3 expression were substantially reduced by S1P (Fig. 6a–d). S1P works in a dose-dependent manner (Fig. 6e–g). JTE-013 reversed the inhibition of neutrophil spontaneous apoptosis induced by S1P (Fig. 6h–j). These data suggest that S1PR_2_ is responsible for S1P-extended neutrophil lifespan.

### Gα_i/o_, ERK, and p38 MAPK signaling pathways participate in the S1PR_2_-mediated switch of neutrophil apoptosis to NETosis

The S1PRs coupling to different G-α subunits allow S1P to exert its influence on numerous pathways, such as nuclear factor-κB, phosphatidylinositol-3-kinase/Akt, and mitogen-activated protein kinase (MAPK) signaling pathway^20^. Short lifespan and developmental state of neutrophil preclude transfection or transduction. Pharmacological inhibitors offer a unique opportunity to study the signaling pathways involved in neutrophils.

**Fig. 7:**
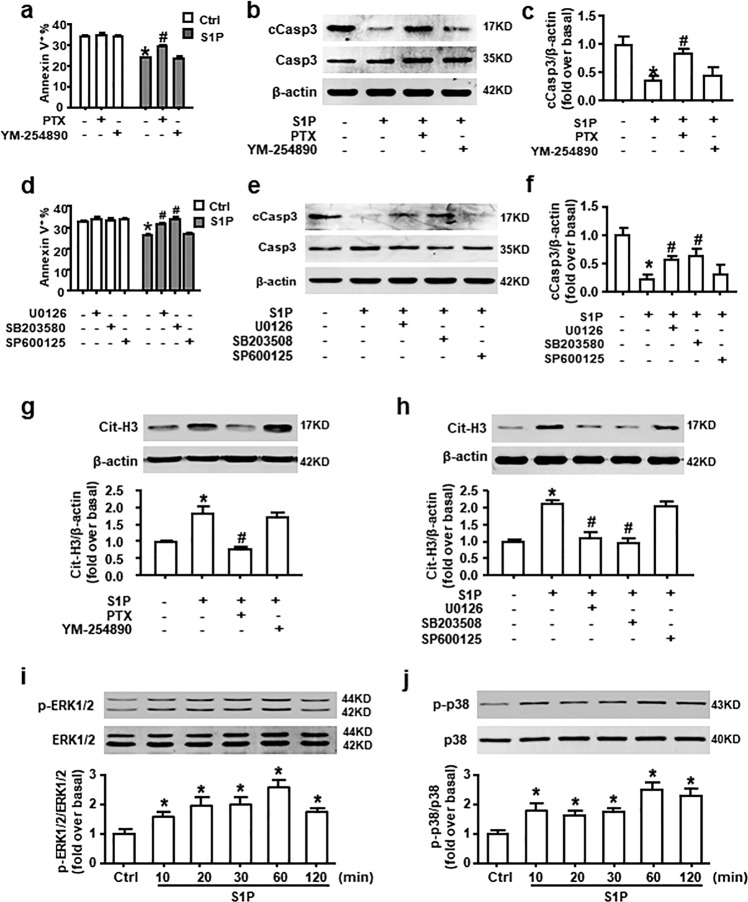
Gα_i/o_/ERK and p38 MAPK signaling pathways are involved in the switch of apoptosis to NETosis induced by S1P in vitro. **a**–**c** Neutrophils were pre-incubated with or without PTX (Gα_i/o_ inhibitor; 10 ng/mL), YM-254890 (Gα_q_ inhibitor; 10 mM), **d**–**f** U0126 (ERK inhibitor; 5 μM), SB203580 (p38 MAPK inhibitor; 5 μM), or SP600125(JNK inhibitor; 5 μM) before neutrophils were exposed to S1P (1 μM) for 8 h. Annexin V and PI staining were used to detect the apoptotic rate. Protein expression of cCasp3 and Casp3 was measured by western blot. **g**, **h** S1P-induced Cit-H3 protein level with or without PTX, YM-254890, U0126, SB203580, or SP600125 pretreatment was examined by western blot. **i**, **j** Phosphor-ERK1/2, total ERK1/2, phosphor-p38, and total p38 expression in neutrophil after S1P treatment was measured by western blot. Data are presented as the mean ± SEM (*n* = 6 per condition in three experiments). One-way ANOVA was used in **i**, **j**, two-way ANOVA was used in **a**, **c**–**d**, and **f**–**h**. *P < 0.05 vs. control. ^#^*P* < 0.05 vs. S1P-treated alone.

First, BM neutrophils were pretreated with PTX (Gα_i/o_ inhibitor) or YM-254890 (Gα_q_ inhibitor) to identify which Gα protein was involved in the effects of S1P. We found that S1P-induced delay of neutrophil apoptosis was prevented by PTX, while it had no effect of its own. YM-254890 did not influence this process (Fig. 7a–c). Then, to further explore the role of MAPK signaling pathways in S1P-induced delay of apoptosis, we pretreated neutrophils with U0126 (ERK inhibitor), SB203580 (p38 MAPK inhibitor), or SP600125 [JNK (c-Jun N-terminal kinase) inhibitor]. Inhibition of ERK or p38 MAPK, but not JNK, signaling pathways reversed S1P-induced reduction of apoptotic cell number and cCasp3 expression (Fig. 7d–f).

After that, we wondered if the same signaling pathways were involved in the S1P-induced NETosis. The augment of Cit-H3 expression was lessened by PTX, U0126, and SB203580 (Fig. 7g, h). Simultaneously, stimulation with S1P led to an increase of phosphor-ERK and phosphor-p38 at the protein level (Fig. 7i, j). Taken together, these results demonstrate that S1P redirects neutrophil apoptosis to NETosis through Gα_i/o_, ERK, and p38 MAPK signaling pathways.

### NADPH oxidase-derived ROS are downstream of ERK and p38 MAPK and participate in the effect of S1P on switch of apoptosis to NETosis

**Fig. 8:**
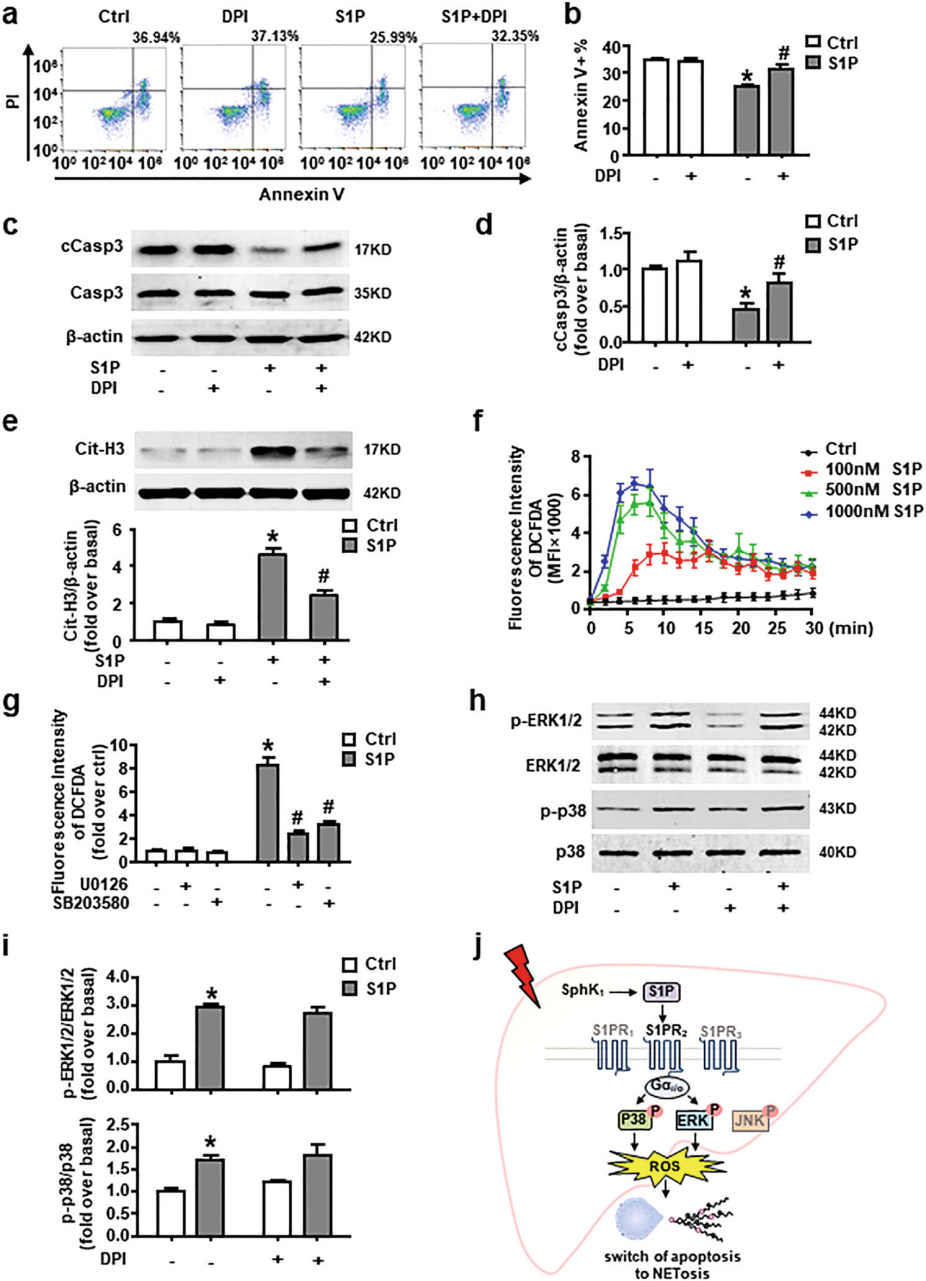
NADPH oxidase-derived ROS are downstream of ERK and p38 MAPK and involved in S1PR_2_-mediated switch of apoptosis to NETosis. **a**, **b** Apoptotic rate of S1P-stimulated neutrophil at 8 h with or without DPI (10 µM) pretreatment was detected by Annexin V and PI staining. Representative FACS plot and quantification of Annexin V^+^ cells are shown. **c**, **d** Protein expression of cCasp3 and Casp3 in neutrophil cultured in S1P pre-incubated with or without DPI was measured by western blot. **e** Cit-H3 protein level in S1P-acivated neutrophil pretreated with or without DPI. **f** ROS production was measured using the DCFDA dye and the fluorescence intensity was detected every 2 min after neutrophils cultured with or without S1P gradients. **g** Pretreated with U0126/SB203580 or not, S1P-induced ROS production was measured. **h**, **i** Phosphorylation level of ERK1/2 and p38 MAPK in S1P-activated neutrophil with or without DPI pretreatment was detected by western blot. **j** Scheme of neutrophil activation mediated by S1PR_2_ during liver injury. Data are presented as the mean ± SEM (*n* = 6 per condition in three experiments). Two-way ANOVA was used. *P < 0.05 vs. control. ^#^*P* < 0.05 vs. S1P-treated alone.

Nicotinamide adenine dinucleotide phosphate (NADPH) oxidase-derived ROS in neutrophil not only cause tissue damage, but also function as signaling mediators inside the cell. They play an essential role in the regulation of NETosis^13^. NADPH oxidase-derived ROS have also been reported to act as a direct molecular switch for redirecting neutrophil apoptosis to NETosis^11,35,36^. Here we showed that the selective NADPH oxidase inhibitor diphenyleneiodonium (DPI) reversed S1P-induced reduction of Annexin V^+^ cell number and cCasp3 expression (Fig. 8a–d). Simultaneously, the results of western blot analysis presented that DPI dramatically blocked S1P-induced Cit-H3 expression (Fig. 8e). To further confirm the involvement of ROS in this shift, we measured the ROS production in a DCFDA plate reader assay. We observed that ROS production sharply increased after S1P treatment in a dose-dependent manner (Fig. 8f). ERK or p38 MAPK inhibitors blocked the ROS production (Fig. 8g), while DPI failed to suppress the phosphorylation of ERK or p38 MAPK in neutrophil stimulated by S1P (Fig. 8h, i). These data suggest that S1P-induced switch of apoptosis to NETosis depends on NADPH oxidase-derived ROS, which is downstream of ERK and p38 MAPK.

## Discussion

Our investigation suggests that NET formation play an important role in the early stage of fatty liver injury. A positive correlation exists between marker of neutrophil activation and SphK1/S1P in the MCDHF-treated mice. In vitro, we find that redirection of neutrophil apoptosis to NETosis mediated by S1PR_2_ depend on Gα_i/o_, ERK, p38 MAPK, and ROS signaling pathways. In vivo, administration of S1PR_2_-siRNA to MCDHF-fed mice significantly attenuates NET formation and the development of liver injury. A concept diagram is drawn to summarize our major findings (Fig. 8j).

Neutrophils are the most abundant leukocyte type in the human circulation and have traditionally been considered to play key roles in acute inflammatory responses. Recently, their contribution to chronic inflammation has been appreciated^2,4,27,32,37^. In this study, we observe that neutrophil infiltration occurred at 7 days and obviously downregulated after 14 days in MCDHF-treated liver. Similarly, in a clinically relevant mouse model of NASH, an influx of neutrophils in livers is found at 5 weeks and returns to baseline by 12 weeks^27^. In contrast, during acute liver inflammation induced by focal necrotic injury, neutrophil numbers inside the injury site reached peak levels at 12 h, but were almost entirely absent by 48 h^38^. During I/R liver injury, NET formation could be detected after 6 h of reperfusion. By contrast, we did not observe NET formation in the liver until 7 days after treatment with MCDHF diet. These phenomena indicate that there exist different neutrophil infiltration and activation patterns between acute and chronic liver diseases.

NET has been confirmed as an important activator in noninfectious inflammatory conditions^39^. Inhibition of NET formation has been recently proved to be protective in mouse models of lupus, cardiac infarction, and thrombosis^40–42^. Here, after identifying that NETs are also formed in MCDHF-induced fatty liver injury, DNase I injection is performed to investigate the role of NETs in this pathogenesis. The elimination of NETs significantly reduces inflammatory cytokine production, ALT and GGT levels, and degree of inflammation and fibrosis on histology. These beneficial effects indicate that NETs greatly contribute to the development of MCDHF-induced liver injury. It has been reported that NETs induce cell death of hepatocytes and stimulate Kupffer cells to express inflammatory cytokines and chemokines^5^, which is indispensable for the onset of inflammatory response^43,44^. Furthermore, NETs are confirmed to activate macrophage for cytokine production, while it is a major source of inflammation factors leading to amplification of the inflammation^45^. Whereas due to the complicated composition of NETs, further study is needed to verify the detailed mechanism of its effect on liver injury.

Depending on the conditions that neutrophils are involved in, they may undergo different types of cell death, such as apoptosis, necrosis, necroptosis, autophagy, NETosis, and pyroptosis. Under normal condition, neutrophils have a short half-life in the circulation^34^. Nevertheless, neutrophil lifespan is extended as a result of exposure to pro-inflammatory mediators at sites of inflammation^32,33,46–48^. NETosis is a novel form of cell death without DNA fragmentation and allows neutrophils to fulfill their capacity beyond lifespan. Previously, most of researches have put attention on the enhanced chemotaxis of neutrophil induced by S1P^49–52^. Here, we first reported that S1PR_2_ mediates the elevation of Cit-H3 level and reduction of cCasp3 level in neutrophils activated by S1P in vitro. Moreover, S1PR_2_ knockdown successfully attenuates NET formation in MCDHF-induced liver injury.

S1P acts as a crucial inflammatory mediator and is emerging as an accomplice in liver diseases^19,22,23,53^. The rapidly increasing prevalence of NASH requires novel therapeutic approaches based on further understanding of its pathogenesis to stop disease progression to fibrosis, cirrhosis, and cancer^54–56^. Therefore, we take advantage of MCDHF diet to induce fatty liver injury in mice. The hepatic histology of MCDHF-fed mice is characterized with lipid accumulation, and significant increase in inflammation and fibrosis^23^. Our published research has confirmed that administration of S1PR_2_ antagonist relieves the extent of inflammation and fibrosis in this model^23^. Here, we notice that NET formation is markedly attenuated in mice with S1PR_2_ knockdown. In view of that NET formation occur in the early stage and plays an important role in liver injury, it can be a therapeutic target of S1PR_2_ blockage in the treatment of chronic liver injury.

In addition, the important role of ERK, p38 MAPK, and ROS signaling pathways is confirmed in this process. ROS have been detected to modulate neutrophil destiny, as a second messenger in a variety of cell death-related signaling pathways^11,13,57^. Inhibitors of ERK and p38 MAPK block S1P-mediated ROS production, while DPI does not prevent phosphorylation of ERK and p38 MAPK, implying that they are upstream of the ROS. This agrees with previous findings that ERK phosphorylates the NADPH oxidase component p47phox^24^. Besides, further study is essential to explore the downstream of ROS in mediating the fate of neutrophil.

In conclusion, we identify the critical role of S1PR_2_ in the mechanism of S1P-induced switch from apoptosis to NETosis. Our results reveal the possibility that S1PR_2_ knockdown can alleviate inflammatory response during fatty liver disease via reducing NET formation.
